# Perioperative teriparatide in adult spinal deformity: a retrospective comparative cohort study

**DOI:** 10.1097/MS9.0000000000005321

**Published:** 2026-07-10

**Authors:** Negin Safari Dehnavi, Sadegh Bagherzadeh, Diego Soto Rubio, Ashley Rogers Lorimer, Dana Saleh, Gautam Rao, Ryan Screven, Gersham Rainone, Puya Alikhani, Mohsen Rostami

**Affiliations:** a Department of Neurosurgery, Shariati Hospital, Tehran University of Medical Sciences, Tehran, Iran; b Department of Neurosurgery, Brain and Spine, Morsani College of Medicine, University of South Florida, Tampa, Florida, USA

**Keywords:** adult spinal deformity, postoperative complications, spinal fusion, teriparatide

## Abstract

**Background:**

Adults with low bone mineral density (BMD) face higher risks of implant loosening, pseudarthrosis, and junctional complications following long-segment spinal deformity reconstruction. Teriparatide (TPTD), an anabolic agent that enhances bone formation, may improve fixation strength and fusion quality. This study compared outcomes between patients with low BMD who received perioperative TPTD and those with normal BMD undergoing long-segment adult spinal deformity (ASD) surgery.

**Methods:**

In a retrospective comparative cohort study, 50 consecutive adults who underwent thoracolumbar fusion with pelvic fixation between June 2022 and January 2024 were included. Patients were classified into two cohorts: normal BMD (*n* = 30) and anabolic users (*n* = 20), who received daily subcutaneous TPTD (20 µg) starting 3 months before surgery and continued for three months after. Clinical outcomes were assessed with the Oswestry Disability Index (ODI) and visual analog scale (VAS) for pain. Radiographic alignment parameters and complication rates were also compared.

**Results:**

Baseline characteristics were similar, except for lower BMD in the TPTD group (mean T-score −1.7 vs 0.95, *P* < 0.001). Both groups showed marked improvements in ODI (−42.5 vs −40.0, *P* = 0.447) and VAS pain (−5.1 vs −6.7, *P* = 0.282). Sagittal alignment correction was comparable between groups, and there were no significant differences in rates of pseudarthrosis (45.0% vs 33.3%), proximal junctional kyphosis (10.0% vs 16.6%), or reoperation (30.0% vs 16.6%). No medication-related adverse effects were noted.

**Conclusions:**

Perioperative TPTD in low-BMD patients demonstrated similar clinical, radiographic, and complication outcomes to those of patients with normal bone density undergoing long-segment ASD reconstruction. These results contribute to the role of structured bone-health optimization in ASD surgery but cannot be attributed to TPTD alone.

## Introduction

Adult spinal deformity (ASD) reconstruction often requires long-segment thoracolumbar constructs with pelvic fixation[1]. Durable arthrodesis must be achieved despite age-related declines in bone mineral density (BMD) and the high prevalence of osteoporosis, which weaken pedicle screw stability and impair fusion biology^[^2,3^]^. Reduced BMD, assessed with dual-energy X-ray absorptiometry (DEXA), is associated with higher risks of pseudoarthrosis, implant failure, and junctional problems, including proximal junctional kyphosis (PJK) and proximal junctional failure (PJF)^[^4,5^]^. Screening and treatment remain underused in practice, with national data showing low rates of preoperative DEXA testing and limited adoption of anti-osteoporosis medication among candidates for complex deformity surgery^[^6^]^.

HIGHLIGHTS

- Perioperative teriparatide equalized outcomes in low vs. normal bone mineral density adult spinal deformity patients.
- Pain, disability, and spinopelvic alignment improved similarly in both cohorts.
- Proximal junctional kyphosis, pseudarthrosis, hardware failure, and reoperation rates were comparable.
- Findings support integrating anabolic therapy into bone health optimization pathways.

Teriparatide (TPTD), recombinant parathyroid hormone PTH 1-34, is an anabolic therapy that stimulates osteoblast activity and improves cancellous bone microarchitecture. In lumbar fusion cohorts, TPTD has been associated with higher radiographic fusion and lower pedicle screw loosening than bisphosphonates or routine care, and pooled analyses report favorable effects on fusion indices^[^7–9^]^. Duration appears important. In geriatric lumbar fusion, treatment for at least 6 months has been linked to a higher 1-year union rate on computed tomography (CT) and more favorable trajectories for pain on the visual analog scale (VAS) and, at times, disability on the Oswestry Disability Index (ODI)[10].

Evidence specific to long-segment ASD is emerging. Observational and multicenter analyses report that, in osteoporotic or osteopenic patients, preoperative or perioperative TPTD can yield complication profiles and patient-reported outcomes (PROs) that approximate those of patients with normal bone density, with selective improvements in pain and reductions in reoperation or symptomatic pseudarthrosis in some cohorts^[^11,12^]^. In a randomized study of osteoporotic adult deformity surgery, perioperative TPTD reduced severe junctional failure and was well tolerated compared with an active antiresorptive control[13]. These data align with reviews that identify low BMD as a consistent contributor to junctional complications and advocate integrated bone health pathways combining patient-specific alignment targets with pharmacologic optimization^[^4,14^]^.

This study aimed to assess whether there was a difference in clinical, radiographic, and complication outcomes between adults with low BMD undergoing long-segment thoracolumbar ASD reconstruction and receiving perioperative TPTD. The study was intended to test the comparability of outcomes as opposed to isolating the independent causal effect of TPTD, since the allocation of treatment reflected real-world bone-health status and clinical decision-making. This is a cohort study that has been reported in accordance with the STROCSS guidelines[15].

## Methods

### Study design and setting

This is a retrospective comparative cohort study conducted at a single tertiary spine referral center. The patients who had multilevel thoracolumbar reconstruction performed to treat symptomatic ASD were identified and studied in two groups (normal BMD, *n* = 30; and anabolic users, *n* = 20), respectively, depending on their bone status and perioperative therapy. Approved by the Institutional Review Board, the requirement for informed consent was waived due to the retrospective design and use of de-identified data. The study was conducted in accordance with Good Clinical Practice and included consecutive cases from June 2022 to January 2024.

### Participants

Adults (18 years and older) with symptomatic ASD, based on SRS-Schwab criteria[16] who planned to undergo posterior instrumented fusion across the thoracolumbar junction with pelvic fixation, were eligible. The analytic cohort included only the patients who had completed the planned 6-month perioperative treatment period. Exclusion criteria included a history of metabolic bone disease other than osteoporosis, severe renal insufficiency, active malignancy, medical or psychosocial factors likely to prevent adherence to perioperative therapy, and loss to follow-up. Prior surgery history was defined as any previous lumbar or thoracolumbar decompression or fusion surgery performed before the index ASD reconstruction. Therefore, patients with prior surgery history were considered to be undergoing revision ASD reconstruction at the index operation, whereas patients without prior surgery history were considered primary ASD reconstruction cases. Baseline demographics, such as age, sex, body mass index, Modified Frailty Index 5 (mFI-5), smoking history, prior spine surgery, and attributes related to the index surgery, were prospectively collected.

### Interventions and perioperative care

All patients received posterior segmental pedicle-screw and rod constructs to correct thoracolumbar deformity. The operating surgeon based his choice of interbody support, osteotomy type, use of supplemental rods, and pelvic fixation on the pattern of deformity and his mechanical stability goals.

Perioperative bone-health optimization was consistently carried out, including nutritional support and smoking-cessation counseling when appropriate. Vitamin D and calcium were routinely prescribed according to age-specific daily requirements[17]. Patients in the anabolic users cohort had low BMD and were treated with TPTD (Forteo; recombinant PTH 1–34), 20 µg subcutaneously once daily, starting 3 months before surgery and continuing for 3 months after, together with vitamin D and calcium supplementation. Patients with normal BMD did not receive anabolic therapy.

TPTD adherence was assessed through medication reconciliation, medication history, and clinical follow-up documentation. All patients who participated in the anabolic cohort had completed the planned 6-month perioperative TPTD course; those who did not receive it were excluded from the analytic cohort. Adverse events that were medication-related, as well as early discontinuation, were documented when they occurred.

### Outcomes

The key clinical outcomes were changes in disability and pain between baseline and follow-up, measured with the ODI and a VAS for the average of radicular and axial pain. The spinopelvic alignment parameters, as well as mechanical and medical complications, were considered secondary outcomes. Radiographic alignment included sagittal vertical axis (SVA), pelvic tilt (PT), lumbar lordosis (LL), and pelvic incidence minus lumbar lordosis (PI-LL) discrepancy, measured before surgery and at follow-up. Mechanical complications of the index surgery up to the latest possible follow-up included PJK, PJF, hardware failure, rod fracture, screw loosening or pullout, pseudarthrosis, and any unexpected reoperation. Hardware failure was defined radiographically and included any findings related to the implant, such as peri-screw lucency suggestive of screw loosening, screw pullout, or rod fracture; no symptoms were required to make this definition. Pseudarthrosis was identified as a radiologic diagnosis of nonunion and did not necessitate any clinical manifestations. The new proximal junctional sagittal Cobb angle between the upper instrumented vertebra (UIV) and UIV + 2 of at least 10 degrees, along with an increase of at least 10 degrees, was considered PJK. PJF was defined as PJK that involved either a vertebral or implant failure, progressive subluxation, a neurologic deficiency, or required a revision surgery[16]. Medical events consisted of venous thromboembolism, urinary tract infection, pneumonia, sepsis, packed red cell transfusion demand, and length of stay in the hospital.

### Imaging and measurements

Preoperative bone health was measured by DEXA of the lumbar spine and/or hip, where possible. The standardized standing full-spine long-cassette radiographs were taken. Spinopelvic measurements were performed by trained reviewers who did not participate in clinical care and were not made aware of the treatment group during the measurement sessions.

The final follow-up radiological evaluation of pseudarthrosis was conducted using CT scans. The criteria included a lack of continuous bridging trabecular bone across the proposed area of fusion or radiolucency around implants, such as peri-screw lucency, which is suggestive of loosening and nonunion. Radiologic factors were evaluated once by a surgeon who had completed a spine fellowship program. Formal inter-rater reliability was not calculated, as the primary outcomes of interest were clinical outcomes (ODI and VAS), and radiologic factors were secondary outcomes measured by a single reviewer.

### Follow-up schedule

The assessments were made at baseline and at the final postoperative follow-up. The analytic follow-up used the most recent available visit for each endpoint; the mean follow-up duration was 382 days in the normal BMD cohort and 607 days in the anabolic cohort. Early postoperative visits occurred as part of routine clinical care and were captured when available, with supplemental encounters recorded when clinically indicated. Compliance and adverse events were assessed at every touchpoint.

### Bias

We considered selection bias through consecutive case inclusion and measurement bias via standardized imaging protocols with blinded radiographic reviewers; potential confounding was addressed by prespecifying covariates (age, sex, BMI, DEXA T-score, prior surgery, mFI-5, interbody count, total fused levels, and UIV).

### Statistical analysis

Means with standard deviation or medians with interquartile range were used to summarize continuous variables by distribution, and counts with percentages were used to summarize categorical variables. The Shapiro–Wilk test was used to check normality. Between-group comparisons involved independent-samples *t*-tests for normally distributed variables or Mann–Whitney *U* tests when the requirements could not be met, and categorical variables were compared with the help of chi-square tests or Fisher’s exact tests in cases where the expected values were less than five. The analysis of multivariate logistic regression was conducted to determine the factors that were independently associated with mechanical complications, which were defined as PJK or hardware failure. The number of covariates in the model was limited based on the events-per-variable principle due to the limited sample size and to prevent overfitting in the model. This resulted in the selection of three clinically relevant variables to be included in the final model. Adjusted odds ratios with 95% confidence intervals (CIs) were estimated to determine the relationship between each variable and the incidence of mechanical complications. Statistical significance was determined to be *P* < 0.05. Missing data were addressed, and all analyses were performed in GraphPad Prism version 8.

## Results

### Cohort and perioperative characteristics

Fifty adults met the inclusion criteria and were analyzed: normal BMD (*n* = 30) and anabolic users (*n* = 20). Of the 20 patients who received perioperative TPTD, all 20 (100%) completed the 6-month course as stipulated in the study. None of the patients stopped therapy prematurely, and no adverse effects related to medications required discontinuation. Groups were broadly comparable at baseline, except for expected differences in bone health and body mass. The mean age was 67.8 vs. 63.6 years (*P* = 0.178), with 60% vs. 40% male (*P* = 0.165). BMI was higher in the Normal BMD cohort (32.8 vs. 25.8 kg/m^2^, *P* < 0.001). The mean DEXA T-score was 0.95 vs. −1.7 (*P* < 0.001). Prior spine surgery (60% vs. 45%, *P* = 0.297), smoking exposure (pack-years 9.8 vs. 10.5, *P* = 0.466), and mFI-5 frailty (2.5 vs. 2.0, *P* = 0.311) were similar. Index constructs had a comparable number of interbodies per case (2.9 vs. 2.5, *P* = 0.436), with a non-significant trend toward more fused levels in the anabolic cohort (7.18 vs. 8.78, *P* = 0.079). The mean follow-up was 382 vs. 607 days (*P* = 0.187). Baseline and operative characteristics are summarized in Table 1.

**Table 1:** Baseline and operative characteristics by group (normal bone marrow density vs teriparatide users).

	Normal BMD (*n* = 30)	Anabolic teriparatide (*n* = 20)	*P*-value
Age, years	67.8 (5.9)	63.6 (12.6)	0.178
Male	18 (60)	8 (40)	0.165
BMI (kg/m^2^)	32.8 (5.3)	25.8 (4.3)	<0.001*
DEXA (T-score)	0.95 0.78	−1.7 0.89	<0.001*
History of spine surgery	18 (60)	9 (45)	0.297
Smoker pack-years	9.8 (18.0)	10.5 (13.5)	0.466
mFI-5	2.5 (1.8)	2.0 (1.02)	0.311
Interbody fusion levels	2.9 (1.5)	2.5 (1.8)	0.436
Total fused levels	7.18 (2.5)	8.78 (3.5)	0.079
UIV			0.405
Upper thoracic T1–T5	3	4	
Lower thoracic T9–T12	13	10	
Upper lumbar L1–L2	14	6	
Follow up, days	382 (221)	607 (524)	0.187

Values are mean ± SD or *n* (%) unless stated otherwise. *P*-values reflect between-group comparisons (normal BMD vs. anabolic teriparatide). BMD, bone mineral density; BMI, body mass index; DEXA, dual-energy X-ray absorptiometry; mFI-5, 5-item modified frailty index; UIV, upper instrumented vertebra. Bold style indicates statistical significance (*P*-value < 0.05).

### Patient-reported outcomes

**Figure 1.**
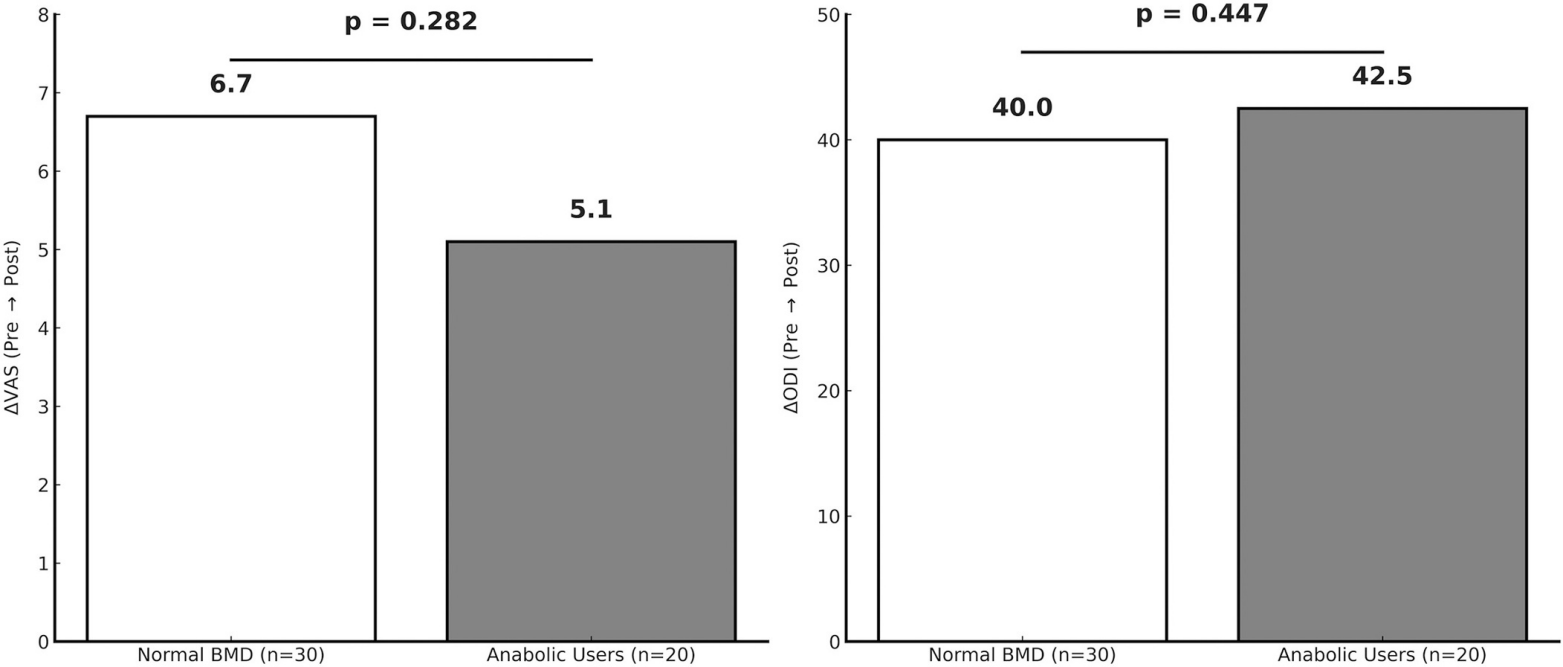
Patient-reported outcomes: mean change (Δ) in VAS pain and ODI from preoperative baseline to final follow-up for Normal BMD vs teriparatide users; error bars = SD. BMD, bone mineral density; ODI, Oswestry Disability Index; VAS, visual analog scale.

Both cohorts experienced large, clinically meaningful improvements in pain and disability from baseline to final follow-up. VAS back pain improved from 8.1 to 1.6 in normal BMD and from 6.4 to 1.3 in anabolic users (within-group *P* < 0.001 for both); the magnitude of improvement (ΔVAS −6.7 vs. −5.1) did not differ between groups (*P* = 0.282). ODI improved from 55.3 to 15.3 vs. 57.7 to 15.2 (within-group *P* < 0.001 for both); the between-group change (ΔODI −40.0 vs. −42.5) was not significant (*P* = 0.447). Full PRO data appear in Table 2 and are illustrated in Figure 1.

**Table 2:** Patient reported outcomes and radiographic parameters: pre–post changes and between-group comparisons (normal bone marrow density vs teriparatide users).

	Normal BMD (*n* = 30)	Anabolic teriparatide (*n* = 20)	*P*-value		
VAS					
Preoperative	8.1 (5.9)	6.4 (2.1)	0.662		
Postoperative	1.6 (1.8)	1.3 (1.2)	0.815		
Δ	−6.7 (4.6)	−5.1 (2.3)	0.282	<0.001*	<0.001*
ODI					
Preoperative	55.3 (8.6)	57.7 (7.8)	0.474		
Postoperative	15.3 (3.7)	15.2 (4.2)	0.954		
Δ	−40.0 (10.0)	−42.5 (9.8)	0.447	<0.001*	<0.001*
PT °					
Preoperative	25.2 (8.6)	28.2 (9.3)	0.312		
Postoperative	19.8 (8.2)	20.0 (6.8)	0.521		
Δ	−5.4 (9.08)	−8.2 (8.16)	0.528	<0.001*	<0.001*
LL °					
Preoperative	31.1 (13.5)	33.6 (18.6)	0.893		
Postoperative	45.1 (12.7)	51.5 (10.4)	0.185		
Δ	+14.0 (11.5)	+17.9 (16.3)	0.422	<0.001*	<0.001*
C7PI SVA mm					
Preoperative	10.8 (4.7)	10.4 (4.5)	0.322		
Postoperative	7.9 (4.9)	5.9 (3.1)	0.252		
Δ	−2.9 (4.2)	−4.6 (3.5)	0.492	<0.001*	<0.001*

Preoperative and postoperative values are shown as mean ± SD. Δ denotes change from preoperative to postoperative. Within-group *P*-values (improvement over time) and between-group *P*-values (Normal BMD vs Anabolic teriparatide) are provided. VAS, visual analog scale; ODI, Oswestry Disability Index; PT, pelvic tilt; LL, lumbar lordosis; SVA, sagittal vertical axis; C7PL, C7 plumb line; Δ, change from preoperative to postoperative. Bold style indicates statistical significance (*P*-value < 0.05).

### Radiographic alignment

**Figure 2.**
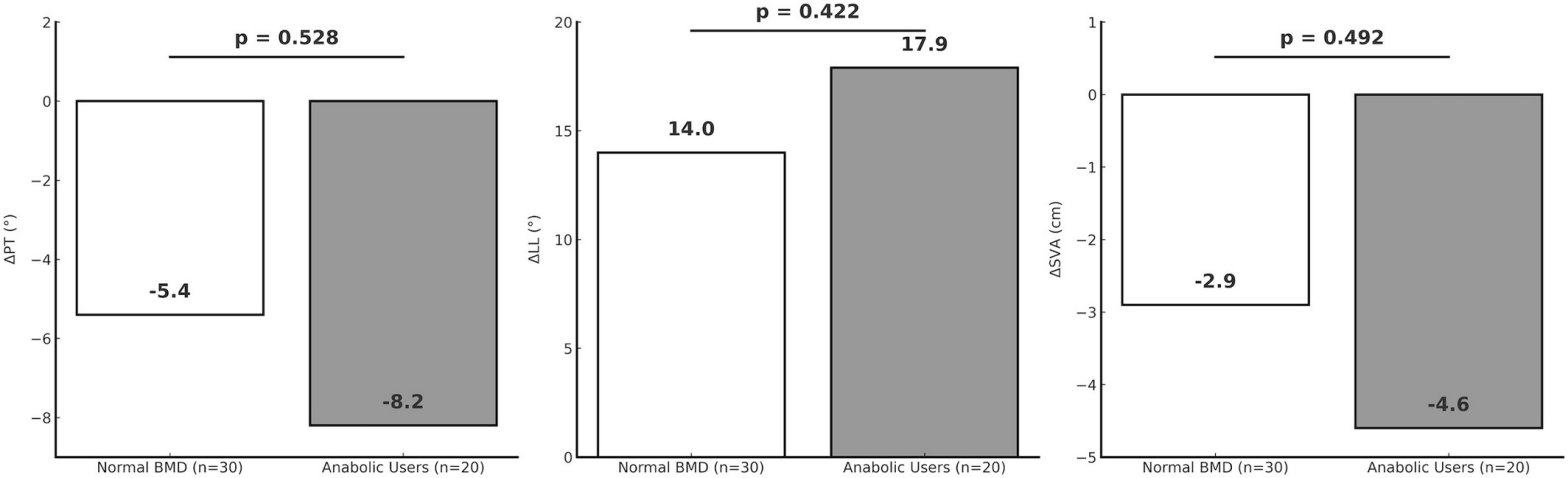
Radiographic alignment: mean change (Δ) in PT (°), LL (°), and SVA (mm) from preoperative baseline to final follow-up for normal BMD vs teriparatide users; error bars = SD. PT, pelvic tilt; LL, lumbar lordosis; SVA, sagittal vertical axis.

Spinopelvic parameters improved significantly within each cohort, with no between-group differences in the magnitude of correction. The mean change in PT was −5.4° vs. −8.2° (*P* = 0.528), LL increased by +14.0° vs. +17.9° (*P* = 0.422), and SVA decreased by −2.9 mm vs. −4.6 mm (*P* = 0.492). Radiographic outcomes are reported in Table 2 and depicted in Figure 2.

### Complications and re-operations

Rates of junctional, mechanical, and medical complications were similar between groups. PJK occurred in 16.6% vs. 10.0% (*P* = 0.687), with no cases requiring revision. Any reoperation occurred in 16.6% vs. 30.0% (*P* = 0.310). Hardware failure was 40.0% vs. 60.0% (*P* = 0.248); rod fracture 9.9% vs. 15.0% (*P* = 0.672); screw pullout 6.6% vs. 5.0% (*P* > 0.999); and pseudarthrosis 33.3% vs. 45.0% (*P* = 0.553). Medical complications occurred in 43.3% vs. 30.0% (*P* = 0.387), including UTI, pneumonia, and sepsis 3.3% vs. 10.0% (*P* = 0.556), and PE/DVT 16.6% vs. 20.0% (*P* > 0.999). Mean packed red blood cell transfusion requirement was 1.8 vs. 2.0 units (*P* = 0.796), and hospital length of stay was 8.6 vs. 7.8 days (*P* = 0.519). Complication and utilization data are detailed in Table 3.

**Table 3:** Complications and resource utilization by group (normal bone marrow density vs teriparatide users).

	Normal BMD (*n* = 30)	Anabolic teriparatide (*n* = 20)	*P* value
PJK	5 (16.6)	2 (10.0)	0.687
PJK requiring revision	0 (0)	0 (0)	>0.999
Any reoperation	5 (16.6)	6 (30.0)	0.310
Any medical complication	13 (43.3)	6 (30.0)	0.387
UTI, pneumonia, sepsis	1 (3.3)	2 (10.0)	0.556
PE, DVT	5 (16.6)	4 (20.0)	>0.999
pRBC units	1.8 (1.84)	2.0 (2.21)	0.796
Any hardware failure	12 (40.0)	12 (60.0)	0.248
Screw pullout	2 (6.6)	1 (5.0)	>0.999
Rod fracture	3 (9.9)	3 (15.0)	0.672
Pseudarthrosis	10 (33.3)	9 (45.0)	0.553
HLOS	8.6 (3.7)	7.8 (2.7)	0.519

Values are mean ± SD or *n* (%) unless stated otherwise. PJK was defined as a proximal junctional sagittal Cobb angle between UIV and UIV + 2 of ≥10° and an increase of ≥10° from baseline; PJF as PJK with vertebral or implant failure, progressive subluxation, new neurologic deficit, or need for revision. PJK, proximal junctional kyphosis; PJF, proximal junctional failure; UTI, urinary tract infection; PE, pulmonary embolism; DVT, deep vein thrombosis; pRBC, packed red blood cells; HLOS, hospital length of stay.

Of the 50 patients studied, 31 experienced mechanical complications. Three clinically significant covariates (length of follow-up, BMI, and history of previous spine surgery) were retained in the final multivariate logistic regression model to reduce overfitting. The adjusted analysis did not identify any of the included variables as independent predictors of mechanical complications. The final model had an approximate ratio of events-per-variable of about 10, which indicates sufficient model structure and minimizes the risk of overfitting. Table 4 summarizes the complete multivariable findings.

**Table 4:** Multivariate logistic regression for baseline factors important regarding the association with mechanical complications (proximal junctional kyphosis or hardware failure).

Variable	Adjusted odds ratio (95% CI)	*P*-value
Prior surgery history	0.633 (0.184–2.177)	0.468
Follow-up length	1.001 (0.999–1.003)	0.272
BMI	1.020 (0.914–1.137)	0.728

BMI, body mass index; CI, confidence interval.

## Discussion

### Principal findings

In both exposure cohorts, pain and disability improved significantly, and PT, LL, and the SVA were also corrected. Between-group differences in PRO improvement and alignment change were not statistically significant. The occurrence of PJK, hardware-related events, pseudarthrosis, reoperation, and medical complications did not differ significantly between groups. These results indicate a substantial clinical improvement. They are in line with reports that structured perioperative channels can stabilize a situation despite skeletal vulnerability^[^1,2,5^]^.

### Relation to randomized and comparative evidence

The identified complication profile and PRO course are consistent with the randomized and comparative analyses of perioperative TPTD in osteoporotic patients. In adults with osteoporosis undergoing an ASD reconstruction, the difference in PJF, pain, and health-related quality of life was found to be less with perioperative TPTD as opposed to active antiresorptive control[13]. In a multicenter study, osteoporotic patients who were treated with TPTD exhibited similar 2-year results as patients with normal BMD and reduced reoperation and symptomatic pseudarthrosis rates compared to osteopenic patients[11]. Other comparative cohorts on deformity and degenerative fusion support an association between TPTD exposure and better fixation biology or lower complication rates in populations with low BMD^[^18–20^]^.

The time of exposure is pertinent to the biologic effect. At least 6 months in geriatric lumbar fusion surgery correlated with a higher 1-year CT union and a better outcome in terms of pain and, occasionally, disability, which guides preoperative planning and postoperative care methods[10]. Radiographic benefits of TPTD over control have been demonstrated by meta-analytic syntheses in osteoporotic cohorts of fusion and have favored pooled effects on fusion indicators^[^8,9^]^. There is early mechanistic clinical evidence of less pedicle screw loosening with TPTD than with bisphosphonate following an anterior lumbar fusion, which supports the plausibility of better fixation in bone of poor quality^[^7,21^]^. Cost-utility modeling also indicates that preoperative TPTD may be cost-effective in some selected osteoporotic individuals when nonunion and revision reductions are achieved^[^22,23^]^.

### Junctional biomechanics and bone quality

The combination of construct length, target alignment, preservation of soft tissue, and the quality of vertebral bone at the uppermost instrumented vertebra causes junctional complications. Low BMD and osteoporosis are found to be constant risk factors enhancing proximal junctional issues, and the countermeasures include optimizing bone health, positioning correctly with age, and selective cranial end augmentation^[^1,4^]^. The causal factor of bone quality in that multifactorial framework is supported by evidence of reduced PJF with perioperative TPTD[13].

### Screening and implementation

The evidence-based practice of screening and pharmacologic optimization is still not in place for complex deformity reconstruction candidates, even though they support it. A national study revealed that eligibility for evaluation increased over time, but the use of preoperative DEXA was about 16%, and the perioperative treatment of osteoporosis was rare, which highlights the need to implement changes in systems of care[6]. Multidisciplinary perioperative care, incorporating anabolic and antiresorptive agents, the preference for organized bone health pathways, and opportunistic imaging surrogates in the absence of deformity-centric DEXA limitations, are the topics of consensus and practice reviews^[^2,5,14^]^.

### Safety, duration, and value

In spine cohorts, perioperative TPTD demonstrates a reasonable safety profile, with minimal medication-related adverse events in randomized and observational designs^[^13,20^]^. The optimal period has not yet been established; clues lean toward a longer period of union support in weak bone, followed by maintenance antiresorptive therapy to maintain the gains^[^11,24^]^. Comprehensive reviews of anti-osteoporosis therapy suggest that efficacy-safety trade-offs by class can be used to select and sequence agents individually in perioperative courses[9]. Pathway evaluations and formal cost-effectiveness assessments are still on the agenda to explain value in ASD programs[22].

### Implications for surgical practice

With respect to surgeons intending long-segment thoracolumbar reconstruction in adults with low BMD, the available evidence supports early recognition of skeletal frailty, specific perioperative pharmacotherapy, and alignment calculation that takes into account age-specific goals. Perioperative TPTD has the potential to aid fixation biology and decrease the probability of destructive junctional failure in patients with the anticipation of significant symptomatic enhancement through cohort groups under modern reconstruction and optimization guidelines.

### Limitations

A number of limitations are to be recognized. First, this retrospective comparison cohort is prone to confounding by indication: low-BMD patients were given perioperative TPTD, whereas normal-BMD patients were not. Therefore, the research considers the comparability of outcomes as opposed to the causal independent effect of TPTD. Second, the groups differed in BMI and years of follow-up, and irregular timing of complications constrained standard time-to-event or 1-year analyses. Though adjusted regression, which included BMI, length of follow-up, and previous spine surgery, did not find independent predictors of the mechanical complications, the small sample size limits precision. Third, the pseudarthrosis and hardware-related events were defined based on broad radiographic definitions in a high-risk population of deformities and were not always symptomatic or revision-requiring. Fourth, the formal inter-rater reliability was not computed. Lastly, larger prospective or randomized studies that compare treated and untreated low-BMD patients are required to make inferences about causation.

## Conclusion

In this retrospective comparative cohort study, adults with low BMD undergoing complex spinal deformity surgery and managed with perioperative TPTD achieved clinical, radiographic, and complication outcomes comparable to those of patients with normal bone density. Both groups demonstrated significant improvements in pain, disability, and spinal alignment, without statistically significant differences in complications or reoperations. These findings support the importance of integrating bone-health optimization into ASD care, while recognizing that the study design does not establish an independent causal effect of TPTD.

## Data Availability

Data and analysis code are available from the corresponding author upon reasonable request.
